# How Artificial Intelligence Can Advance Electrochemical Science and Identify Water Molecule Orientation on Platinum Electrodes

**DOI:** 10.1021/acs.jpcc.5c08343

**Published:** 2026-01-28

**Authors:** Yitao He, Jiří Červenka

**Affiliations:** † Department of New Energy Science and Engineering, School of Energy and Environment, Anhui University of Technology, Ma’anshan 243002, China; ‡ Department of Thin Films and Nanostructures, FZU − Institute of Physics of the Czech Academy of Sciences, Cukrovarnická 10/112, Prague 6 162 00, Czech Republic

## Abstract

Electrochemistry lies at the heart of modern energy technologies, yet connecting atomic-level insights to macroscopic performance remains an enduring challenge. Quantum-based simulations, such as density functional theory, have illuminated many fundamental processes, but their reach is limited by the complexity of real electrochemical environments. Bridging these scales requires a new conceptual framework that can expose the hidden connections between theory and experiment. Here, we argue that the thoughtful integration of artificial intelligence (AI) can transform electrochemical research by unifying theory, experiment, and data-driven inference. AI-assisted frameworks can accelerate convergence between computation and experiment, revealing hidden physical relationships and enabling closed-loop discovery. Realizing this vision will require developing transparent, interpretable AI models that earn the same scientific trust as human reasoning, unlocking deeper understanding and innovation across the electrochemical sciences.

## Graphical abstract

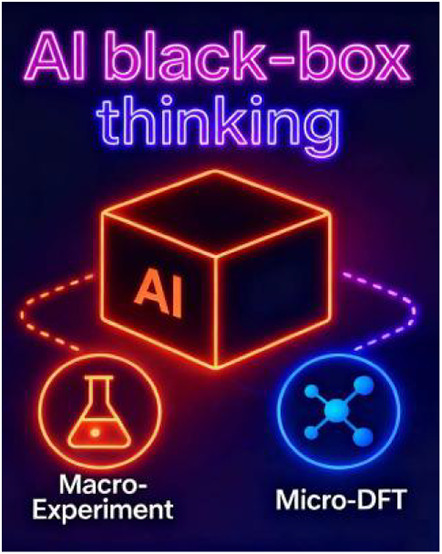

Electrochemistry forms the fundamental basis for a wide range of technologies central to modern energy conversion and storage systems,^1,2^ including batteries,^3^ electrocatalysis,^4,5^ electrochemical sensors,^6–9^ water treatment,^10^ electroplating,^11^ and biomedicine.^12–14^ Understanding and optimizing these systems require insight into their complex interfacial reactions, which occur across multiple length and time scales. In this context, quantum-based computational methods, such as density functional theory (DFT),^15^ are commonly employed to model realistic electrochemical systems and elucidate reaction mechanisms at the atomic scale.^16,17^ However, as highlighted by Govindarajan et al.,^18^ a significant gap remains between microscopic simulations and macroscopic electrochemical systems.^19,20^ Achieving accurate multiscale modeling is challenging because numerous physical and environmental factors must be considered simultaneously, many of which are difficult to incorporate explicitly without introducing extensive approximations.^21,22^ Bridging this gap requires a new paradigm that can learn the missing physics between quantum-level simulations and macroscopic reality.^23^

## Opportunities for AI in Electrochemistry

Modern artificial intelligence (AI) tools,^24^ particularly since the emergence of large language models (LLMs) such as ChatGPT,^25^ have profoundly transformed research workflows and information processing across disciplines.^26–28^ AI tools can also serve as valuable assistants for researchers,^7^ helping to streamline literature reviews, refine manuscripts, analyze complex data sets, and even provide inspiration for new research directions.^29,30^ However, researchers should more proactively explore how to integrate AI tools to accelerate advancements in fundamental research.^31–33^ Here, we demonstrate that embracing AI thoughtfully could open new frontiers in electrochemical research.

AI tools encompass a wide range of models, including neural networks,^34^ which have long been applied in chemistry,^35^ for instance, in chemometrics.^36,37^ However, in 2022, the models that have truly generated a worldwide impact are LLMs and mature market products like ChatGPT.^24,38^ Even after more than two years, many researchers remain skeptical about whether these tools can provide reliable scientific insights, particularly in electrochemistry,^39^ which is deeply rooted in experimental validation and traditional equation-based theoretical models. However, as mentioned before, significant gaps remain between typical macroscopic models and quantum-level microscopic models. For example, DFT calculations are commonly used to interpret experimental phenomena or to support conclusions drawn from macroscopic, equation-based models (e.g., continuum electrochemical models, Nernst–Planck equations, and Butler–Volmer kinetics). Yet, the connection between these scales is nontrivial, and discrepancies in magnitude are often ignored, with DFT calculations frequently cited solely as mechanistic evidence.

Despite significant progress in this field, solutions to these problems remain largely infeasible. We previously highlighted this issue in a review paper^40^ and had sought a solution until we attended the tutorial “*Artificial Intelligence in Electrochemistry*” at the 76th Annual Meeting of the International Society of Electrochemistry, delivered by Jan Rossmeisl from the University of Copenhagen. During the presentation, a slide outlined three stages of progress in theoretical electrochemistry: from equation-based models to DFT models and now to AI models. At that moment, it became clear that AI tools could bridge macroscopic and microscopic models and potentially provide a pathway to solutions that were previously unattainable. Even though this example is included solely as contextual background and does not constitute the scientific basis of the framework proposed in this work, eventually, AI tools could operate at a higher-dimensional level by harnessing advanced mathematical and statistical methods.^41^

Here, we present an example in which an AI system, represented by ChatGPT, is employed to refine or calibrate DFT results by leveraging experimental data as a reference, thereby establishing a micro–macro bridge. Because the model’s internal reasoning is not known a priori, we refer conceptually to it as “AI black-box thinking” (AI-BBT), as illustrated in Scheme 1. This is analogous to the black-box elements used in traditional equivalent-circuit analyses of electrochemical impedance spectroscopy (EIS) data. Rather than replacing DFT predictions, the AI learns from systematic deviations between theoretical and experimental behavior, inferring a correction function that accounts for missing physical phenomena. The AI-BBT serves as an inference layer that integrates these two domains, uncovering the hidden relationships and linking realistic electrode behavior with atomistic modeling. In this work, the term “AI-BBT” refers to the AI-assisted exploratory process by which candidate model structures are proposed, rather than to the final models themselves. We do not claim transparency into ChatGPT’s internal reasoning during this exploratory step and therefore treat it as a black box at the level of cognition. Importantly, all candidate models are subjected to explicit, physics-based selection criteria, and only models that are predictive, stable, and physically interpretable are retained with the experimental data. Thus, while AI-assisted exploration may be opaque, the final selected model is fully transparent and interpretable and is judged solely by its scientific usefulness.

**Scheme 1:**
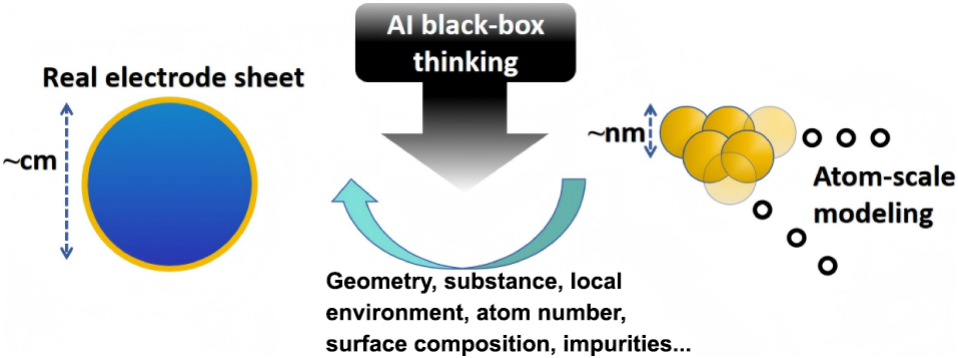
Conceptual Schematic Illustrating how AI Black-Box Thinking Bridges Macroscopic and Microscopic Electrochemical Models

## AI as a Tool for Connecting Macroscopic and Atomistic Data

In a typical electrochemical system, an aqueous electrolyte meets a working electrode to form a dynamic interface, where charge transfer, ion transport, and solvent reorganization occur. EIS probes this interface across frequencies, resolving process-specific time constants and separating overlapping phenomena. In this study, we use EIS—together with DRT analysis^42^—as a quantitative window on interfacial dynamics and as a testbed to demonstrate how AI-BBT calibrates theory to experiment and reveals field-driven, polarity-dependent effects.

First, a combination of experimental measurements and DFT calculations was conducted to obtain the fundamental data. In a three-electrode open-cell configuration, two platinum (Pt) pin electrodes served as the working and counter electrodes, while a Hg/HgO electrode was employed as the reference. Pure deionized water, without any salts, was used as the electrolyte. The EIS was performed under various applied biases (vs the OCP: 0, 0.1, 0.2, 0.4, and 0.6 V, ±) within the frequency range of 0.1–10^6^ Hz. The corresponding experimental Nyquist plots are presented in Figure 1A, and the distribution of relaxation times (DRT) plots (Figure 1B) were derived from the EIS data using F. Ciucci’s DRT analysis tool.^43^ Two dominant peaks appear in the DRT spectra, each reflecting a characteristic relaxation time of interfacial H_2_O. Because our measurement window (0.1–10^6^ Hz) is far below the THz range of intramolecular vibrations, we assign the high-frequency peak (short τ) to fast dipolar reorientation and the low-frequency peak (long τ) to slower configurational rearrangement involving hydrogen-bond reorganization and partial dissociation/adsorption of water. Therefore, here, we consider only the high-frequency peak that can exclude the dissociation processes.

**Figure 1:**
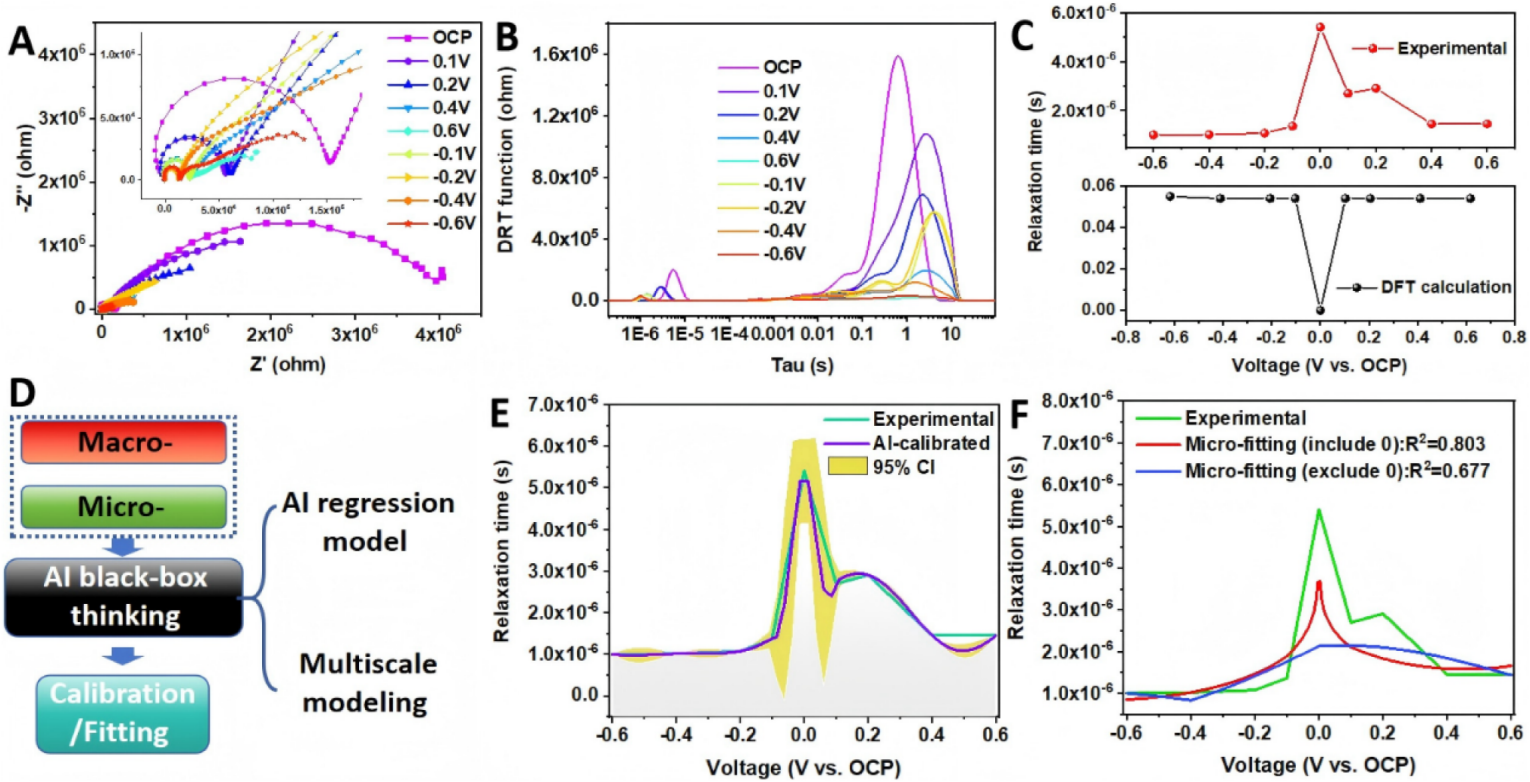
(A) Experimental Nyquist plots obtained at different applied potentials. (B) Corresponding DRT plots derived from the impedance data. (C) Comparison between experimental and DFT-calculated relaxation times as a function of voltage. (D) Conceptual schematic of the AI-bridged framework (“AI black-box thinking”), showing how microscopic (from DFT) and macroscopic (from EIS) data are integrated through AI regression and multiscale modeling. (E) AI-calibrated relaxation–time curve with 95% confidence interval (CI), demonstrating agreement between calculation and experiment. (F) Parametric microfitting of DFT-derived data with and without inclusion of the OCP point, showing correlation coefficients of *R* ^2^ = 0.803 and *R* ^2^ = 0.677, respectively.

Because DFT models cannot perfectly reproduce real electrochemical environments, increasing the number of atoms dramatically increases the computational cost. Even the addition of just two H_2_O molecules requires a high-performance computing system. Therefore, to emphasize the key role of the AI-assisted tool, a simplified model containing a single H_2_O molecule was constructed and optimized in Gaussian 09 ^44^ (DFT, B3LYP/6-311G­(d)) under comparable *x*-directional electric fields (0, ±0.0004, ±0.0008, ±0.0016, and ±0.0024 a.u., where one atomic unit (a.u.) of electric field corresponds to approximately 5.142 × 10^11^ V m^–1^. Considering reasonably that the thickness of the electric double layer (EDL) in pure water is about 0.5 nm,^45^ an applied potential of 0.1 V approximately corresponds to an electric field strength of 0.0004 a.u.) Then, transition states (TS) were computed for all bias conditions referenced to 0 V. Using transition-state theory (Eyring equation), the activation barriers were converted to rate constants, from which the molecular relaxation times were obtained.

Figure 1C compares the experimental (high-frequency peak) and calculated relaxation times. The two differ by roughly 3 orders of magnitude (μs experimentally vs ms theoretically), which we attribute to model idealizations in the calculations and potential experimental uncertainties. To reconcile these scales, we apply an AI-assisted calibration that links DFT-derived barriers to experimental relaxation times, yielding consistent, data-driven corrections.

Finally, when the commercial version of GPT-5 was employed, the AI-BBT framework provided two rapid solutions to this problem within an astonishing 30 s. Figure 1D conceptually illustrates the structure of this AI-bridged framework, referred to as AI-BBT. In this process, microscopic data from DFT calculations and macroscopic data from EIS measurements were input into the AI-BBT, which automatically identified suitable mathematical and machine-learning models. Two complementary models were generated: a regression model and a multiscale model. Among them, the AI regression model serves as a bridge between microscopic (DFT level) and macroscopic (experimental) domains, enabling calibration and fitting across scales without predefined equations.

In this workflow, AI serves as a black-box exploration assistant to propose and screen candidate model structures, while the final selected model remains fully transparent and is judged solely by its physical interpretability and usefulness. In practice, the search procedure consisted of providing ChatGPT with the full data set, the explicit scientific objective, and a physically constrained set of candidate descriptors, and using it as an AI-assisted reasoning tool to iteratively propose, screen, and simplify candidate model forms, while final model selection was performed based on predefined physical and performance criteria. The first calibration model is a nonparametric Gaussian process regression, a Bayesian machine learning method that learns a nonlinear mapping from microscopic descriptors derived from DFT calculations to macroscopic relaxation behavior while providing uncertainty estimates. The second model is a parametric, physics-informed linear regression that extracts mechanistic meanings from the data through interpretable coefficients. It is worth noting that neither approach relies on neural networks, as AI-BBT is the optimal choice. Instead, they represent two complementary forms of machine learning—nonparametric Bayesian learning for flexible mapping and parametric surrogate modeling for physical interpretability.

Ultimately, the primary criterion guiding both descriptor selection and model construction is that the resulting model provides a consistent and physically meaningful description of the experimental data. Within the physics-first, AI-assisted AI-BBT framework, descriptors are retained only if they contribute useful information and improve the model’s ability to capture key experimental trends without introducing unnecessary complexity. In this sense, model and descriptor selections follow a pragmatic yet physically grounded philosophy: the simple, interpretable models that work and yield insights are preferred, analogous to the selection of equivalent circuits in EIS.

### AI Calibration Process Conducted by a Nonparametric AI Regression Model: Determining Microscopic Descriptor

A nonparametric AI model—specifically, a Gaussian Process (GP)^46,47^ —is employed, a probabilistic regression technique that can model nonlinear relationships without assuming a fixed parametric form.^48^ The data set consists of experimental relaxation-time measurements collected over a defined voltage range, paired with DFT-derived microscopic relaxation times from a single molecule. All data are shown in Figure 1. The feature space is low-dimensional and explicitly defined, with no latent variables or automated feature extraction. Experimental noise is treated implicitly within the GP likelihood. Mathematical relationships are expressed as

1 $$\tau_{\exp}=f\left(V,\tau_{\mathrm{calc}},V\tau_{\mathrm{calc}}\right)+\varepsilon$$

where *f* is the latent nonlinear relationship that the GP aims to uncover, and *ε* denotes experimental noise. There are three input variables to capture possible coupling between the electric field and molecular response; among them, *Vτ*_calc_ encodes nonlinear field–molecule coupling and asymmetrical effects. The kernel function (covariance between data points) was chosen by AI as

2 $$k\left(X_{i},X_{j}\right)=\sigma^{2}\exp\left[-\underset{d}{\sum }\frac{{\left(x_{i,d}-x_{j,d}\right)}^{2}}{2\gamma_{d}^{2}}\right]+\sigma_{n}^{2}\delta_{ij}$$

where *σ* ^2^ is a scaling constant that adjusts the overall magnitude of the covariance, *γ*_*d*_ represents length scales for each input dimension $\left(\gamma_{V,\tau_{\mathrm{calc}},V\tau_{\mathrm{calc}}}\right)$ ), determined using automatic relevance determination (ARD),^49,50^ which allows each dimension to have its own scale, reflecting its importance or variability; $\sigma_{n}^{2}\delta_{ij}$ is a small white-noise term added to the diagonal, accounting for measurement noise or model imperfections. The exponential term measures similarity between points across all dimensions *d*. By leveraging its kernel and the observed data, a GP infers a distribution of plausible functions that fit complex nonlinear patterns. It then provides probabilistic predictions, quantifying uncertainty through a confidence interval derived from the underlying covariance structure.

For visualization, a fine voltage grid (−0.6 to +0.6 V, 50 points) was generated. Then, the expected value (mean) *τ̂*_exp_(*V*) = E ­[*f*(*V*)] of the latent function *f*(*V*) was predicted by the GP at each voltage. The square root of the variance $\sigma \left(V\right)=\sqrt{\mathrm{Var}\left[f\left(V\right)\right]}$ represents the uncertainty in the prediction, quantifying the possible deviation of the predicted mean from the true value. The corresponding 95% confidence interval (CI) is given by [*τ̂*_exp_ – 1.96*σ*(*V*), *τ̂*_exp_ + 1.96*σ*(*V*)], implying that there is a 95% probability (under the GP model) that the true value of *τ*_exp_ lies within this range. The resulting prediction curve with mean and uncertainty bands is presented in Figure 1E. The AI model successfully reproduces the experimental voltage-dependent relaxation behavior. The close match between the AI-calibrated and experimental results shows that the GP model has effectively learned the missing physical effects absent in DFT. In this calibration, the GP model uses DFT-derived relaxation times as input features and experimental data as training targets, thereby calibrating the microscopic predictions against the macroscopic reality.

The workflow of GP is shown in Figure 2A. The optimized ARD length scales reveal a clear hierarchy in how the microscopic and macroscopic variables influence the experimental relaxation behavior. Specifically, the ordering $\gamma_{V\tau_{\mathrm{calc}}}>\gamma_{V}>\gamma_{\tau_{\mathrm{calc}}}$ (Table 1) indicates that the GP model responds most sharply to variations in the DFT-derived microscopic relaxation parameter, while the effects of voltage and explicit voltage–barrier coupling become progressively smoother. In a GP, a smaller length scale corresponds to a rapidly varying, highly nonlinear dependence; therefore, the smallest scale, associated with *τ*_calc_, shows that subtle changes in the microscopic reorientation barrier produce pronounced variations in the macroscopic relaxation time. The intermediate voltage scale, *γ*_*V*_, suggests that the applied field contributes a secondary but clearly resolvable modulation of *τ*_exp_, indicating that the role of interfacial dipole alignment and field-induced structural response is important but does not dominate the relaxation dynamics. Meanwhile, in contrast, the very large coupling scale $\gamma_{V\tau_{\mathrm{calc}}}$ implies that the explicit nonlinear interaction term $V\tau_{\mathrm{calc}}$ produces only a weak curvature in the GP latent function.

**Figure 2:**
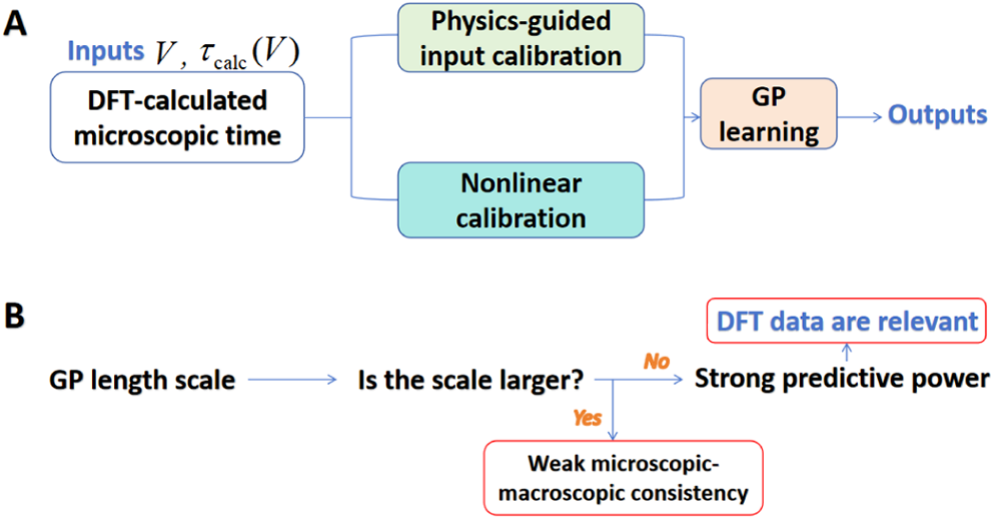
(A) Workflow of the nonparametric AI calibration: DFT-calculated microscopic relaxation times are first preprocessed by physics-guided and nonlinear transformations and then learned by a GP model to generate a calibrated microscopic–macroscopic relationship. (B) A flowchart illustrating how the GP kernel length scale associated with the DFT-calculated barrier is used to evaluate microscopic–macroscopic consistency.

**Table 1:** Fitting Values of Length Scales

GP length scale	Value	Meaning of the value
$\gamma_{V}$	0.246	Moderate scale → sensitivity to voltage
$\gamma_{\tau_{\mathrm{calc}}}$	0.0187	Strongest/smallest scale → highest curvature
$\gamma_{V\tau_{\mathrm{calc}}}$	18.1	Very large → GP barely varies along this dimension

Therefore, as shown in Figure 2B, the much smaller length scale associated with the DFT-calculated relaxation barrier indicates that variations in this microscopic single-molecule descriptor have a strong influence on the experimentally observed relaxation. This behavior reflects a high degree of microscopic–macroscopic consistency: although the DFT data represent the transition-state energetics of an isolated water molecule, the experiment probes the collective interfacial response governed by extended hydrogen-bond networks, dielectric screening, and many-body polarization effects. Consequently, the GP framework does more than interpolate the data—it quantitatively assesses the physical relevance of each microscopic descriptor by learning how sensitively the macroscopic observable responds along each input dimension. In this sense, the GP-derived length-scale hierarchy offers a direct, data-driven measure of how effectively the DFT-calculated barrier carries over to ensemble-level relaxation dynamics.^51^ Moreover, because irrelevant or weakly correlated variables naturally obtain very large GP length scales, the framework also serves as a diagnostic tool for identifying suitable microscopic descriptors. Descriptors with small or moderate scales contribute meaningfully to the macroscopic response, whereas those with extremely large scales are effectively filtered out by the model. In this way, the GP not only evaluates the microscopic–macroscopic consistency of the DFT input but also guides the selection or refinement of physically relevant descriptors (Figure 3).

**Figure 3:**
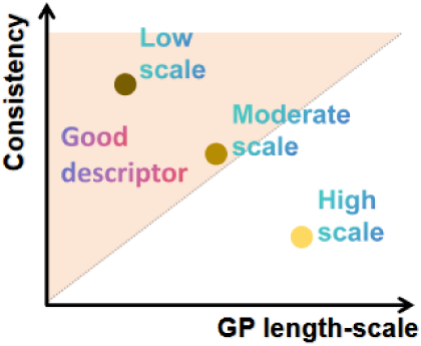
Conceptual illustration of how the GP length scale reflects the microscopic–macroscopic consistency of a descriptor.

Therefore, future work could apply AI-BBT to well-characterized electrochemical benchmarks, such as proton transfer on Pt(111), ion transport in dilute electrolytes, or the Gouy–Chapman–Stern description of the EDL. These systems possess rigorous analytical solutions or high-accuracy simulation references, making them ideal for testing whether AI-BBT faithfully recovers established mechanistic trends and parameter sensitivities. Successful reproduction of known behavior in these cases would both validate the method’s robustness and clarify the scope of problems where AI-BBT delivers genuine physical insight rather than purely correlative descriptions.

### Bridging the Microscopic and Macroscopic via a Multiscale Modeling Framework: Physical Interpretation

At the molecular level, water rotation over Pt is governed by the activation barrier Δ*E*(*V*) obtained from TS calculations. However, in the actual electrochemical interface, many factors, such as hydrogen bonding, electronic screening by the EDL, and collective polarization of thousands (at least) of molecules, accelerate the relaxation dynamics and introduce a nonlinear dependence on the voltage. Therefore, AI-BBT selects a physics-informed machine learning approach called symbolic regression (or multiscale surrogate model) to automatically discover the best mathematical correction function. Although the GP analysis indicates that the microscopic DFT barrier alone shows a limited direct correlation with the experimental relaxation times, this does not undermine the value of the DFT descriptor. Rather, it reflects the intrinsic multiscale nature of the system: the experiment probes a collective, field-driven interfacial response that cannot be captured by single-molecule energetics alone. The symbolic multiscale model does not force DFT to match the experiment; instead, it objectively determines how much of the macroscopic behavior originates from the microscopic barrier and how much arises from field-dependent collective effects. By separating and quantifying these contributions through the parameters, the model provides a physically reliable decomposition of the underlying mechanisms.

The generated model assumes that *τ*_exp_(*V*) arises from *τ*_calc_(*V*) multiplied by a voltage-dependent correction factor Φ­(*V*):

3 $$\tau_{\exp}\left(V\right)=A{[\tau_{\mathrm{calc}}(V)]}^{\alpha }\Phi \left(V\right)$$

with

4 $$\ln\tau_{\exp}=a+\alpha \ln\tau_{\mathrm{calc}}+bV+c\left|V\right|+dV^{2}$$

where *A* (=*e* ^*a*^) is a global scaling constant. *α* is the degree of sensitivity of macroscopic *τ*_exp_ to microscopic *τ*_calc_; if *α* = 0, voltage-dependent terms dominate the correction, whereas *α* = 1 indicates direct proportionality between the two. The *bV* term captures field polarity asymmetry, reflecting how the direction of the electric field (positive or negative potential) affects the relaxation time. The *c*|*V*| term relates to voltage magnitude, accounting for the absolute field strength, while the *dV* ^2^ term represents second-order effects, such as nonlinear EDL effects and dipole reorientation, both of which depend on the square of the applied voltage.

The model maintains a physically interpretable exponential-type structure while allowing the regression to estimate coefficients directly from the data. Descriptor selection was guided by physical relevance to the measured observable. The experimental target is a voltage-dependent relaxation time, while the only microscopic input available from DFT is a single-molecule TS barrier. From electrochemical considerations, the applied voltage *V* influences interfacial dynamics through polarity, field strength |*V*|, and nonlinear field effects. Accordingly, the candidate descriptor set was restricted to {*τ*_calc_, *V*, |*V*|, *V* ^2^}, which separately represent microscopic energetics, field direction, field magnitude, and the lowest-order nonlinear response. This physically motivated restriction ensures that each descriptor captures a distinct mechanism and avoids redundant or opaque variables. Therefore, after constructing the feature matrix **X** = [ln *τ*_calc_, *V*, |*V*|, *V* ^2^] and target vector *y* = In *τ*_exp_, the linear regression was performed on the (**X**,*y*) pair, and predictions were made over a dense voltage grid of 200 points. The workflow of this multiscale symbolic modeling method is shown in Figure 4.

**Figure 4:**
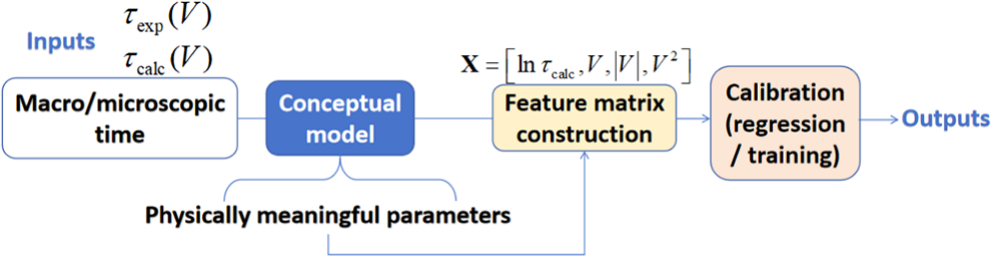
Workflow of the multiscale symbolic modeling: experimental and DFT relaxation times are combined through a physics-informed conceptual model, transformed into a feature matrix, and fitted by using regression-based training to obtain physically meaningful parameters and calibrated predictions.

Because the condition at 0 V (no applied bias) is physically distinct in DFT calculations, two separate models, one excluding and one including the zero-bias point, were constructed. The comparison of these two conditions is shown in Figure 1F (*a* = −13.206, *α* = −0.110, *b* = 0.555, *c* = −2.625, and *d* = 2.272 for the case of including the zero-bias point). The physical insights derived from the symbolic model are summarized in Figure 5. The coefficient *α*, which multiplies *τ*_calc_, represents the degree of correspondence between the experimental and theoretical data, ranging from 0 (unrelated) to 1 (fully related) (Figure 5B). Particularly, if *α* < 0, the macroscopic system behaves inversely to microscopic barriers. The slightly negative value of *α* indicates that the macroscopic relaxation is only weakly inherited from the DFT transition-state barrier, implying that collective interfacial effects dominate the observed dynamics. Thus, the value of −0.110 means that less than approximately 10% of the experimental relaxation trend can be attributed to the DFT barrier.

**Figure 5:**
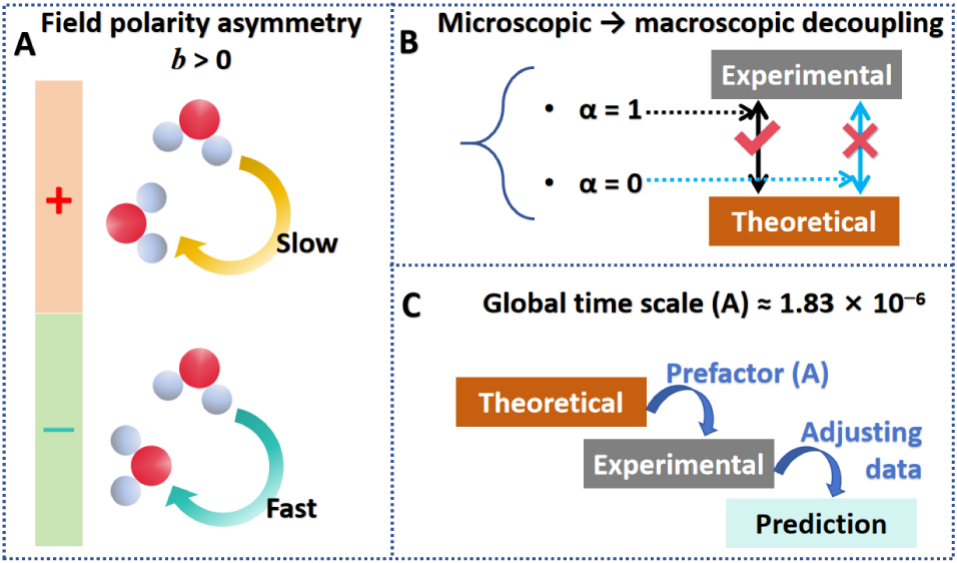
Physical interpretation of the fitted parameters in the model from AI-BBT: (A) field polarity asymmetry governed by *b* > 0, where positive potentials hinder water reorientation while negative potentials accelerate it; (B) microscopic-to-macroscopic decoupling reflected by *α*, showing that *α* → 0 indicates weak inheritance of the DFT-calculated microscopic barrier and strong dominance of interfacial collective effects in the experimental relaxation; (C) global time-scale prefactor *A* bridges the theoretical barrier and experimental dynamics, rescaling the microscopic prediction to match macroscopic measurements and enabling quantitative voltage-dependent predictions.

The positive linear term *b* captures the polarity asymmetry of the interfacial electric field (Figure 5A). A value of *b* = 0.555 indicates that the relaxation time changes by roughly 50% per volt solely due to the direction of the applied potential, consistent with the well-known preference of water molecules to adopt O-down configurations under positive bias and H-down configurations under negative bias. The negative coefficient *c* = −2.625, which multiplies |*V*|, describes the effect of the absolute field strength independent of polarity. Its magnitude reflects the strong dipole-alignment torque exerted by increasing |*V*|, leading to faster orientational relaxation as the interfacial water molecules more readily align with the applied field. In contrast, the positive quadratic coefficient *d* = 2.272 captures second-order nonlinearities arising from dielectric saturation and collective EDL polarization. As |*V*| becomes sufficiently large, these nonlinear polarization effects can counteract the initial acceleration, causing the relaxation time to increase again.

The prefactor *a* reflects the quantum stabilization of the molecular barrier, shifting the intrinsic time scale toward microsecond dynamics. The parameter determines the global time scale, and the extremely small global time scale (*A* = *a* ^–13.206^ ≈ 1.83 × 10^–6^) simply means the orientational stabilization dramatically reduces the absolute time scale predicted by the microscopic model (Figure 5C). The prefactor *A* can be viewed as a conversion factor between two distinct time scales. The DFT-derived relaxation time represents the intrinsic time scale of a single-molecule TS process, whereas the experimental relaxation reflects the collective response of many interfacial molecules acting together. Because these two processes are governed by different physical clocks, systematic rescaling is required. The prefactor *A* provides this rescaling: values of *A* ≪ 1 indicate that collective interactions substantially accelerate the relaxation compared to an isolated molecular event (that is simulation/calculation result), while *A* ≈ 1 implies that the microscopic model already predicts a time scale comparable to the ensemble response. *A* serves as a meaningful scale-bridging quantity rather than a mere fitting correction. Mathematically, *A* rescales the microscopic process to match the macroscopic one; physically, it quantifies how much faster (or slower) the collective process is relative to the microscopic calculative event. Therefore, this shifts the intrinsic relaxation toward faster times, consistent with picosecond–nanosecond water dynamics seen spectroscopically. Together, these terms reproduce the experimentally observed peak at 0 V and its asymmetric suppression away from OCP, demonstrating that *τ*_exp_ is controlled by a competition between molecular reorientation torques and collective interfacial polarization effects.

The accuracy of the model was further evaluated using the coefficient of determination *R* ^2^, which quantifies the proportion of experimental variance captured by the ordinary least-squares regression. These results demonstrate that the inclusion of the zero-bias state ensures continuity across the potential range and enhances the accuracy of the AI-based DFT–experiment bridging model. These results suggest that interfacial water molecules exhibit faster reconfiguration near negatively charged electrode surfaces, consistent with stronger electrostatic interactions between the partially positive hydrogen atoms and the negatively polarized metal surface.

## Comparison of Two Models

Compared with the parametric microfitting models shown in Figure 1F, the nonparametric Gaussian-Process-based AI calibration in Figure 1E provides a more accurate and physically consistent representation of the potential-dependent relaxation behavior. While the analytical models capture the overall physical trend, the nonparametric GP model learns localized nonlinearities and quantifies prediction uncertainty, yielding a higher *R* ^2^ (0.947 vs 0.803). This improvement confirms that interfacial water dynamics are governed by complex, field-dependent correlations beyond simple exponential scaling and that AI can effectively bridge these microscopic and macroscopic regimes through data-driven inference. Importantly, the GP results help clarify the relation between the microscopic and macroscopic regimes: if the microscopic descriptor used in the calculation is sufficiently realistic and captures the essential interfacial physics, then the experimental and theoretical data can be quantitatively reconciled within the AI-BBT framework. Conversely, discrepancies revealed by the GP length scales indicate that the single-molecule DFT model fails to represent the collective behavior of the electric double layer. By adjusting the parameters in the AI-BBT model, we can identify which physical effects are missing or unresolved from the experimental observations and incorporate them into a unified multiscale description. This also suggests that as the microscopic model becomes sufficiently realistic and captures the essential interfacial physics, such as incorporating a larger interfacial water ensemble, explicit hydrogen-bond networks, EDL modeling, and more faithful electrostatic boundary conditions, the discrepancy between experimental and calculated relaxation dynamics can be quantitatively reconciled within the AI-BBT framework. In general, as simulations become more precise, more subtle missing physical factors can be identified. Importantly, AI-BBT does not treat this discrepancy as an error to be eliminated but rather as a source of physical information. The reconciliation is achieved by decomposing the mismatch into a small set of interpretable parameters, which effectively identify and weight the dominant physical contributions as the simulation fidelity improves. As the microscopic model advances infinitely close to reality, one expects systematic shifts in these parameters, reflecting a redistribution of physical weight from phenomenological corrections toward microscopic descriptors. In this way, AI-BBT provides a controlled, physics-informed route to diagnose which interfacial processes dominate the experimental behavior and which aspects of the microscopic model remain incomplete.

In comparison and summary, the nonparametric GP calibration emerges from the AI-BBT process as one of the models automatically proposed by the AI to map the microscopic (DFT) inputs to the macroscopic (experimental) relaxation times. Because the GP is inherently nonparametric, it provides a flexible and unbiased fit to the data but does not yield explicit electrochemically interpretable parameters. To extract mechanistic insight, the AI-BBT process subsequently generated a second model, an interpretable symbolic multiscale expression that embeds physical structure and produces explicit parameters describing how microscopic molecular barriers translate into macroscopic ensemble relaxation dynamics. Thus, within AI-BBT, the GP model represents the best achievable statistical description of the experimental data without physical constraints, while the symbolic model provides a physically interpretable pathway. By comparing the two AI-generated models, we show that AI-BBT simultaneously achieves high predictive accuracy and reveals explicit physical parameters governing microscopic–macroscopic coupling and voltage-dependent interfacial dynamics.

## Conclusions

During the AI calibration, the DFT-predicted and experimentally measured relaxation times are not only quantitatively reconciled but also provide physical insights into interfacial molecular dynamics. The magnitude of the calibration factor (10^–6^–10^–5^) reveals that many effects, such as collective hydrogen-bond and polarization effects, accelerate molecular reorientation by several orders of magnitude relative to single-molecule DFT predictions. The voltage-dependent asymmetry and the OCP-centered peak captured by the AI models indicate that water dipoles reorient nonlinearly under electric fields, with faster alignment near negatively polarized surfaces. Moreover, the variance estimated by the GP model provides a direct measure of configurational fluctuation, which is broad near the OCP and narrow at high voltage, reflecting the transition from disordered to field-ordered interfacial water. Therefore, the AI calibration acts as a bridge that not only corrects DFT predictions but also exposes the collective and nonlinear nature of the dipole dynamics and hidden effects at electrochemical interfaces.

This work also has several limitations. The symbolic model derived by AI-BBT is calibrated using a single experimental observable within a restricted voltage window, and its parameters should, therefore, not be regarded as universal. The microscopic DFT descriptor captures only part of the underlying physics, as it does not include collective hydrogen-bond rearrangements, long-range dielectric screening, or nonlinear EDL restructuring. The symbolic terms correct for these missing contributions but do not replace the full multiscale simulations. Moreover, AI-BBT is designed to extract interpretable structure rather than to provide broad predictive capability, and its conclusions remain bound by the descriptors and data supplied. As an AI-driven approach, the discovered functional form is not guaranteed to be unique; it represents one physically consistent explanation, as inferred from the data. Thus, the main contribution of this work lies in demonstrating how AI can help connect microscopic and macroscopic electrochemical behavior rather than in delivering a fully predictive interfacial model. Despite these limitations, a notable advantage of the AI-BBT framework is its efficiency. Once the DFT barrier data and experimental relaxation measurements are available, the AI calibration can be completed within hours. The entire workflow, from exploratory model generation to final parameter estimation and uncertainty quantification, requires only modest computational resources, yet successfully bridges the microscopic and macroscopic scales that traditionally demand extensive parametrization or iterative simulation. AI-assisted approaches such as AI-BBT can substantially accelerate theoretical–experimental convergence in electrochemistry by mapping complex, multivariate physical relationships directly from data. Looking forward, the scientific questions revealed by electrochemical measurements may be explored more deeply through AI-BBT, which could integrate theoretical computation, experimental data, and AI inference into an intelligent, closed-loop platform for studying interfacial phenomena. At the same time, improving transparency and interpretability will be essential for building trust in AI predictions. The AI tools were applied in a goal-driven and physically constrained manner. The scientific objective and experimental conditions were defined in advance, and the EIS measurements on a real electrochemical interface provided the necessary constraints on both the model inputs and the operating conditions. Within this framework, ChatGPT was used as an AI-assisted exploration and screening tool rather than an autonomous decision-maker. Model selection was based on explicit criteria that combine predictive performance, parameter stability, uncertainty behavior, and physical interpretability. The final model was, therefore, chosen not solely for numerical accuracy but because it is fit for purpose: it reliably describes the experimental behavior while providing physically meaningful insight into the microscopic–macroscopic connection.

## Perspective

The AI-BBT strategy demonstrated in this work provides a generalizable pathway for reconciling microscopic calculations with macroscopic electrochemical observables. Although the present study focuses on water reorientation at a platinum interface, the same black-box thinking framework can be extended to broader electrochemical systems where atomistic and continuum scales are inherently mismatched. Examples include electrocatalytic reaction barriers, ion transport across confined environments, charge compensation in porous electrodes, and solid-electrolyte interphase dynamics. In such systems, conventional single-scale modeling often fails to capture collective effects, interfacial restructuring, and long-range correlations. By allowing AI to autonomously explore both nonparametric, interpretable symbolic models and even neural network models, AI-BBT offers a flexible mechanism for discovering hidden structure in experimental data, identifying the limits of atomistic models, and inferring missing physical descriptors.

Experimental data form the foundation of the AI-BBT framework by defining a physically constrained environment for machine-learning models, while practical applicability serves as the primary criterion for model selection, assisted by AI tools. As real systems become more complex, increasingly realistic simulation models are required that incorporate additional physical factors (e.g., factors A, B, and C). Within this expanded model space, AI tools assist in exploring and selecting candidate models (such as models a, b, and c) that remain both tractable and physically interpretable. On one hand, these models and their associated simulations can be calibrated against experimental data to diagnose which physical mechanisms are missing or underrepresented in the simulations and how such deficiencies manifest in macroscopic behavior. On the other hand, as the database and user community grow, repeated validation across related systems may reveal that certain model forms (e.g., model a) consistently perform well. In such cases, these validated models can be directly transferred to treat similar systems, thereby accelerating the analysis and reducing the need for repeated model construction. Future developments may integrate time-dependent data, operando spectroscopic inputs, or multidescriptor DFT databases, enabling fully data-driven multiscale theories for complex electrochemical interfaces. Ultimately, AI-BBT has the potential to serve as a unifying framework that bridges mechanistic insight and predictive modeling across the entire landscape of electrochemical science.

## Data Availability

The data that support the findings of this study are openly available on Zenodo.org at 10.5281/zenodo.18232300.
